# Supplementary material for: Array of Miniaturized Amperometric Gas Sensors Using Atomic Gold Decorated Pt/PANI Electrodes in Room Temperature Ionic Liquid Films
Source: Sensors (Basel). 2023 Apr 20;23(8):4132. doi: 10.3390/s23084132 (PMC10141993; doi:10.3390/s23084132)

#Supplementary File

Array of Miniaturized Amperometric Gas Sensors Using Atomic Gold Decorated Pt/PANI Electrodes in Room Temperature Ionic Liquid Films

Anifatul Faricha [1], Shohei Yoshida [2], Parthojit Chakraborty [3], Keisuke Okamoto [2], Tso-Fu Mark Chang [3], Masato Sone [3], Takamichi Nakamoto [1, 3*]

[1] Department of Information and Communications Engineering, Tokyo Institute of Technology, 226-8503, Kanagawa, Japan
[2] Department of Materials Science and Engineering, Tokyo Institute of Technology, 226-8503, Kanagawa, Japan
[3] Institute of Innovative Research, Tokyo Institute of Technology, 226-8503, Kanagawa, Japan
*Correspondence: nakamoto.t.ab@m.titech.ac.jp

Lists of supplementary information files:

S1 : The reproducibility of Au_2 clusters decorated Pt/PANI using 3 different IDA electrodes

S2 : The data applying two different modified IDAs (using Pt/PANI/Au_2 as representative) with the same RTIL

S3 : The raw data for Table 4

S4: Schemes of our experimental set up and experiments performed in this research study

S1 : The reproducibility of Au_2 clusters decorated Pt/PANI using 3 different IDA electrodes

(a) IDA 1

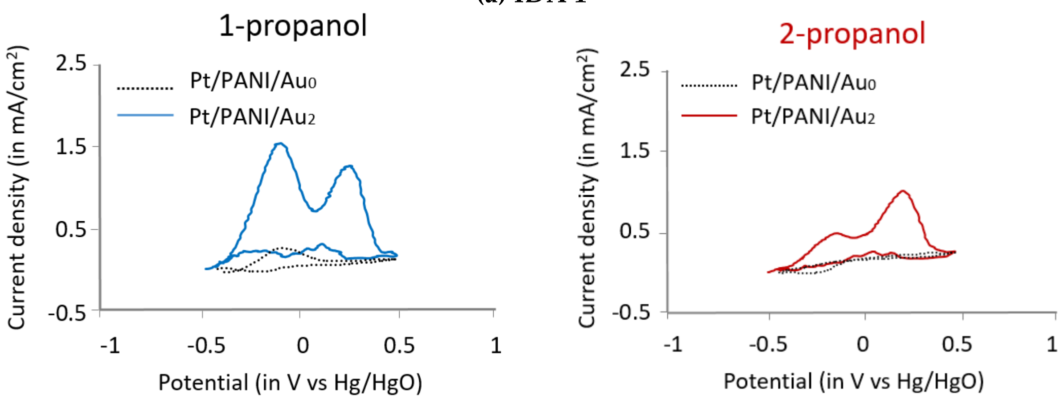

(b) IDA 2

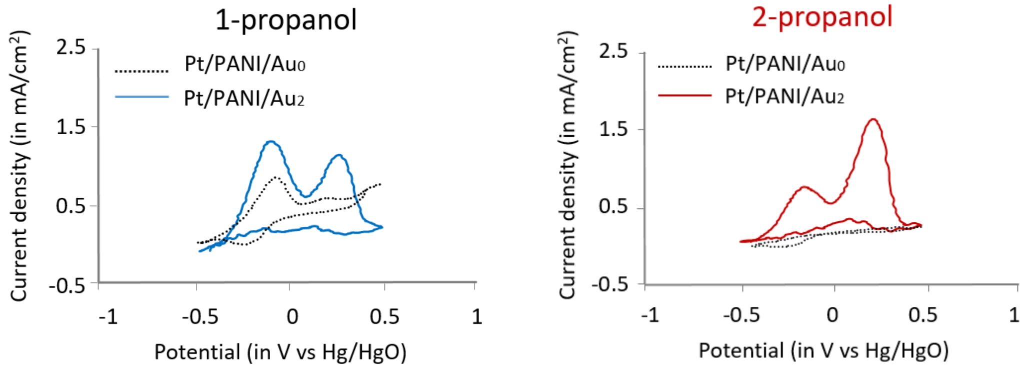

(c) IDA 3

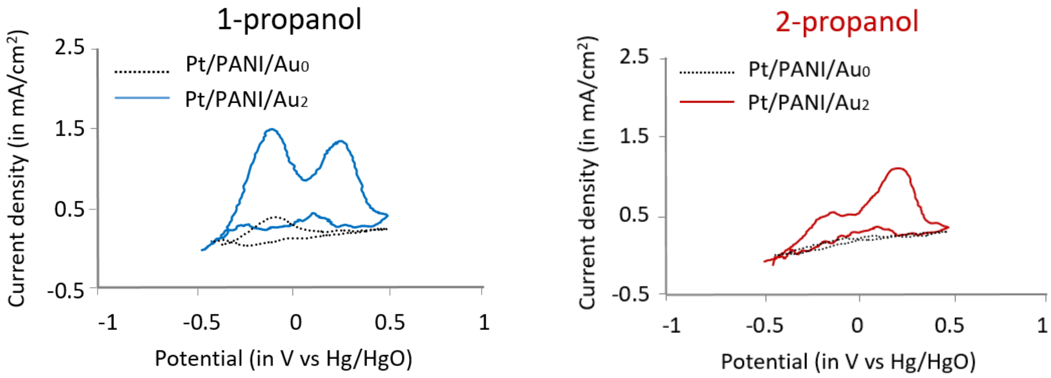

Figure S1. The comparison of electrooxidation from propanol isomers using Pt/PANI/Au_2 and Pt/PANI/Au_0 generated from 3 different IDA electrodes: (**a**) IDA 1; (**b**) IDA 2; (**c**) IDA 3.

S2 : The data applying two different modified IDAs (using Pt/PANI/Au_2 as representative) with the same RTIL

Modified WE : Pt/PANI/Au_2
RTIL : [EMIM][Ac]

RC : 100%

***E*s against Ag/AgCl (V)**	**Analyte**	**Mean in mA/cm²** *standard deviation* **in mA/cm²**	
		**IDA 1**	**IDA 2**
+0.25	1-butanol	7.1<br>*0.1*	7.4<br>*0.141421*
	Isobutanol	7.233<br>*0.0577*	7.25<br>*0.3535*
	2-butanol	6.9<br>*0.1*	6.55<br>*0.777*
+0.5	1-butanol	8.5<br>*0.5*	8.15<br>*0.212*
	Isobutanol	10.33<br>*0.577*	10.55<br>*0.636*
	2-butanol	6.76<br>*0.580*	7.05<br>*0.0707*
+0.9	1-butanol	3.9<br>*0.3605*	4.05<br>*0.007*
	Isobutanol	5.13<br>*0.814*	6.1<br>*0.14*
	2-butanol	4.05<br>*0.3905*	4<br>*0.0001*

* data from IDA 1 and IDA 2 were from 3 and 2 repeated measurements, respectively

Modified WE **: Pt/PANI/Au_2**
RTIL **: [EMIM][Otf]**

RC **: 100%**

***E*s against Ag/AgCl (V)**	**Analyte**	**Mean in mA/cm^2** *standard deviation* **in mA/cm^2**	
		**IDA 1**	**IDA 2**
+0.25	1-butanol	4.667	4.5
		1.527	*2.12*
	Isobutanol	2.667	3
		0.577	*0.707*
	2-butanol	2.667	2
		0.288	*0.0001*
+0.5	1-butanol	3	2.25
		1.3228	*0.3535*
	Isobutanol	0.833	0.5
		0.288	*0.001*
	2-butanol	1.333	1.25
		0.577	*0.3535*
+1	1-butanol	2	1.5
		1	*0.707*
	Isobutanol	0.6	0.5
		0.173	*0.001*
	2-butanol	0.766	0.5
		0.288	*0.00002*

* data from IDA 1 and IDA 2 were from 3 and 2 repeated measurements, respectively

Modified WE : Pt/PANI/Au_2
RTIL : [EMIM][Cl]

RC : 100%

***E*s against Ag/AgCl (V)**	**Analyte**	**Mean in mA/cm^2 *standard deviation* in mA/cm^2**	
		**IDA 1**	**IDA 2**
+0.25	1-butanol	5<br>*1.6667*	5<br>*2.357*
	Isobutanol	10<br>*1.667*	10<br>*2.357*
	2-butanol	1.611<br>*0.1575*	1.79<br>*0.1296*
+0.5	1-butanol	3.222<br>*0.19245*	3.25<br>*0.117*
	Isobutanol	4.111<br>*0.09622*	3.708<br>*0.5303*
	2-butanol	0.00001<br>*0.0000001*	0.0000003<br>*0.0000001*
+1	1-butanol	3.22<br>*0.19245*	3.25<br>*0.11785*
	Isobutanol	4.11<br>*0.09622*	3.7083<br>*0.5303*
	2-butanol	0.0000001<br>*0.0000001*	0.0000001<br>*0.0000001*

* data from IDA 1 and IDA 2 were from 3 and 2 repeated measurements, respectively

S3 : The raw data for Table 4

IDA : Pt/PANI/Au_2
RTIL : [EMIM][Ac]

***E*s against Ag/AgCl (V)**	**Analyte**	**Mean in mA/cm²** / ***standard deviation* in mA/cm²**				
		**0% RC**	**25% RC**	**50% RC**	**75% RC**	**100% RC**
+0.25	1-butanol	0	0.5	3.042	6.0404	7.22
		0	*0.015811*	*0.113225*	*0.151858*	*0.192354*
	Isobutanol	0	0.5	2.592	6.566	7.24
		0	*0.015811*	*0.074632*	*0.084735*	*0.181659*
	2-butanol	0	0.0986	0.0986	0.0986	6.76
		0	*0.014993*	*0.014993*	*0.014993*	*0.439318*
+0.5	1-butanol	0	0.956	2.28	7.006	8.36
		0	*0.092898*	*0.192354*	*0.071274*	*0.415933*
	Isobutanol	0	0.956	8.18	9.072	10.42
		0	*0.092898*	*0.216795*	*0.08167*	*0.531037*
	2-butanol	0	0.956	0.954	6.126	6.88
		0	*0.092898*	*0.094763*	*0.212791*	*0.56952*
+0.9	1-butanol	0	0.956	2.356	2.356	3.96
		0	*0.092898*	*0.202312*	*0.202312*	*0.270185*
	Isobutanol	0	2.776	3.49	3.49	5.52
		0	*0.043932*	*0.074162*	*0.074162*	*0.785493*
	2-butanol	0	0.956	3.47	3.47	4.03
		0	*0.092898*	*0.044721*	*0.044721*	*0.277489*

IDA : Pt/PANI/Au$_0$
RTIL : [EMIM][Ac]

***E*s against Ag/AgCl (V)**	**Analyte**	**Mean in mA/cm^2** *standard deviation* **in mA/cm^2**				
		**0% RC**	**25% RC**	**50% RC**	**75% RC**	**100% RC**
+0.25	1-butanol	0	0.0986	0.0986	0.1018	1.6
		0	*0.014993332*	*0.014993332*	*0.011054411*	*0.547722558*
	Isobutanol	0	0.0986	0.0986	0.0986	1.7
		0	*0.014993332*	*0.014993332*	*0.014993332*	*0.447213595*
	2-butanol	0	0.0986	0.0986	0.0986	1.12
		0	*0.014993332*	*0.014993332*	*0.014993332*	*0.216794834*
+0.5	1-butanol	0	0.954	0.954	0.954	1.34
		0	*0.094762862*	*0.094762862*	*0.094762862*	*0.207364414*
	Isobutanol	0	0.956	0.956	0.956	2.24
		0	*0.092897793*	*0.092897793*	*0.092897793*	*0.450555213*
	2-butanol	0	0.956	0.956	0.956	0.974
		0	*0.092897793*	*0.092897793*	*0.092897793*	*0.043358967*
+0.9	1-butanol	0	0.956	0.956	0.956	1.6
		0	*0.092897793*	*0.092897793*	*0.092897793*	*0.547722558*
	Isobutanol	0	0.956	0.956	0.956	1.7
		0	*0.092897793*	*0.092897793*	*0.092897793*	*0.447213595*
	2-butanol	0	0.956	0.956	0.956	1.12
		0	*0.092897793*	*0.092897793*	*0.092897793*	*0.216794834*

IDA : Pt/PANI/Au2
RTIL : [EMIM][Otf]

***E*s against Ag/AgCl (V)**	**Analyte**	**Mean in mA/cm²** *standard deviation* **in mA/cm²**				
		**0% RC**	**25% RC**	**50% RC**	**75% RC**	**100% RC**
+0.25	1-butanol	0	1.918	2.76	3.46	4.6
		0	*0.08438*	*0.43359*	*0.841427*	*1.516575*
	Isobutanol	0	1.59	2.39	2.66	2.8
		0	*0.654217*	*0.219089*	*0.4219*	*0.570088*
	2-butanol	0	1.154	1.94	2.37	2.4
		0	*0.086487*	*0.089443*	*0.21095*	*0.41833*
+0.5	1-butanol	0	0.94	0.94	2	2.7
		0	*0.089443*	*0.089443*	*0.070711*	*1.036822*
	Isobutanol	0	0.94	0.94	0.94	1
		0	*0.089443*	*0.089443*	*0.089443*	*0.273861*
	2-butanol	0	0.94	0.94	0.94	1.3
		0	*0.089443*	*0.089443*	*0.089443*	*0.447214*
+1	1-butanol	0	0.47	0.47	0.47	1.8
		0	*0.044721*	*0.044721*	*0.044721*	*0.83666*
	Isobutanol	0	0.391	0.391	0.391	0.56
		0	*0.008944*	*0.008944*	*0.008944*	*0.134164*
	2-butanol	0	0.391	0.47	0.47	0.66
		0	*0.008944*	*0.044721*	*0.044721*	*0.250998*

IDA : Pt/PANI/Au_0
RTIL : [EMIM][Otf]

***E*s against Ag/AgCl (V)**	**Analyte**	**Mean in mA/cm^2 *standard deviation* in mA/cm^2**				
		**0% RC**	**25% RC**	**50% RC**	**75% RC**	**100% RC**
+0.25	1-butanol	0	0	0	0	0.0000001
		0	*0*	*0*	*0*	*0.0000001*
	Isobutanol	0	0	0	0	0.0000001
		0	*0*	*0*	*0*	*0.0000001*
	2-butanol	0	0	0	0	0.0000001
		0	*0*	*0*	*0*	*0.0000001*
+0.5	1-butanol	0	0	0	0	0.0000001
		0	*0*	*0*	*0*	*0.0000001*
	Isobutanol	0	0	0	0	0.0000001
		0	*0*	*0*	*0*	*0.0000001*
	2-butanol	0	0	0	0	0.0000001
		0	*0*	*0*	*0*	*0.0000001*
+1	1-butanol	0	0	0	0	0.0000001
		0	*0*	*0*	*0*	*0.0000001*
	Isobutanol	0	0	0	0	0.0000001
		0	*0*	*0*	*0*	*0.0000001*
	2-butanol	0	0	0	0	0.0000001
		0	*0*	*0*	*0*	*0.0000001*

* Three different IDAs were used to check on different days, all three different IDAs showed the same response, and there were no signals of analyte obtained

IDA : Pt/PANI/Au_2
RTIL : [EMIM][Cl]

***E*s against Ag/AgCl (V)**	**Analyte**	**Mean in mA/cm²** *standard deviation* **in mA/cm²**				
		**0% RC**	**25% RC**	**50% RC**	**75% RC**	**100% RC**
+0.25	1-butanol	0	0	0.786667	3.196667	5
		0	*0*	*0.069121*	*0.140633*	*1.666667*
	Isobutanol	0	0	1.596667	4.733333	10
		0	*0*	*0.096032*	*0.383695*	*1.666667*
	2-butanol	0	0	0	0	1.596667
		0	*0*	*0*	*0*	*0.096032*
+0.5	1-butanol	0	0	1.596667	2.15	3.233333
		0	*0*	*0.096032*	*0.442531*	*0.149071*
	Isobutanol	0	0	1.5	3.7	3.9
		0	*0*	*0.096032*	*0.401386*	*0.31*
	2-butanol	0	0	0	0	0.0000003
		0	*0*	*0*	*0*	*0.0000001*
+1	1-butanol	0	0	1.596667	2.15	3.233333
		0	*0*	*0.096032*	*0.442531*	*0.149071*
	Isobutanol	0	0	1.596667	3.766667	3.95
		0	*0*	*0.096032*	*0.401386*	*0.381584*
	2-butanol	0	0	0	0	0.0000001
		0	*0*	*0*	*0*	*0.0000001*

IDA : Pt/PANI/Au_0
RTIL : [EMIM][Cl]

***E*s against Ag/AgCl (V)**	**Analyte**	**Mean in mA/cm^2 / *standard deviation* in mA/cm^2**				
		**0% RC**	**25% RC**	**50% RC**	**75% RC**	**100% RC**
+0.25	1-butanol	0	0	0.7	4	3.233333
		0	*0*	*0.069121*	*5.907387*	*0.149071*
	Isobutanol	0	0	4.266667	4.733333	7.466667
		0	*0*	*5.907387*	*0.383695*	*0.87718*
	2-butanol	0	0	0	0	0.0000001
		0	*0*	*0*	*0*	*0.000001*
+0.5	1-butanol	0	0	1.596667	2.15	3.33333
		0	*0*	*0.096032*	*0.442531*	*0.15*
	Isobutanol	0	0	1.596667	3.766667	3.95
		0	*0*	*0.096032*	*0.401386*	*0.3*
	2-butanol	0	0	0	0	0.0000001
		0	*0*	*0*	*0*	*0.000001*
+1	1-butanol	0	0	1.596667	2.15	3.2
		0	*0*	*0.096032*	*0.442531*	*0.149*
	Isobutanol	0	0	1.5	3.7	3.9
		0	*0*	*0.09*	*0.40*	*0.351584*
	2-butanol	0	0	0	0	0.0000001
		0	*0*	*0*	*0*	*0.000001*

S4: Schemes of our experimental set up and experiments performed in this research study

I. Sensor fabrication

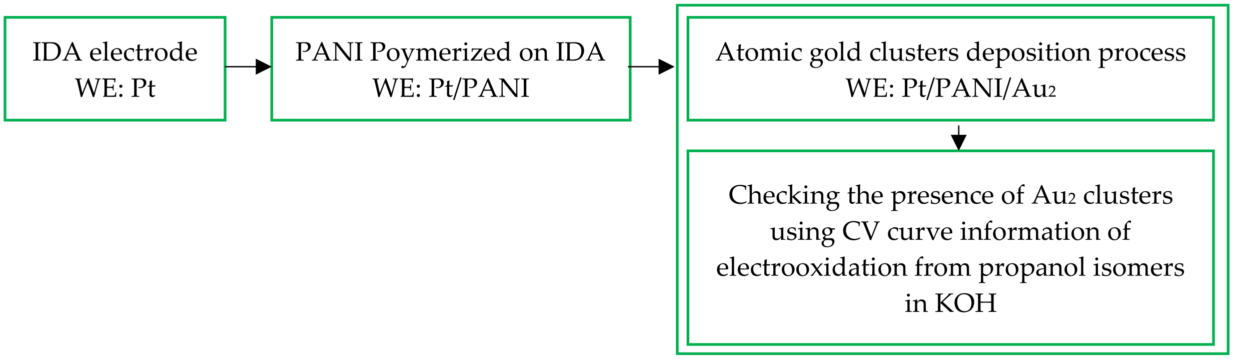

II. Butanol isomers gas measurements

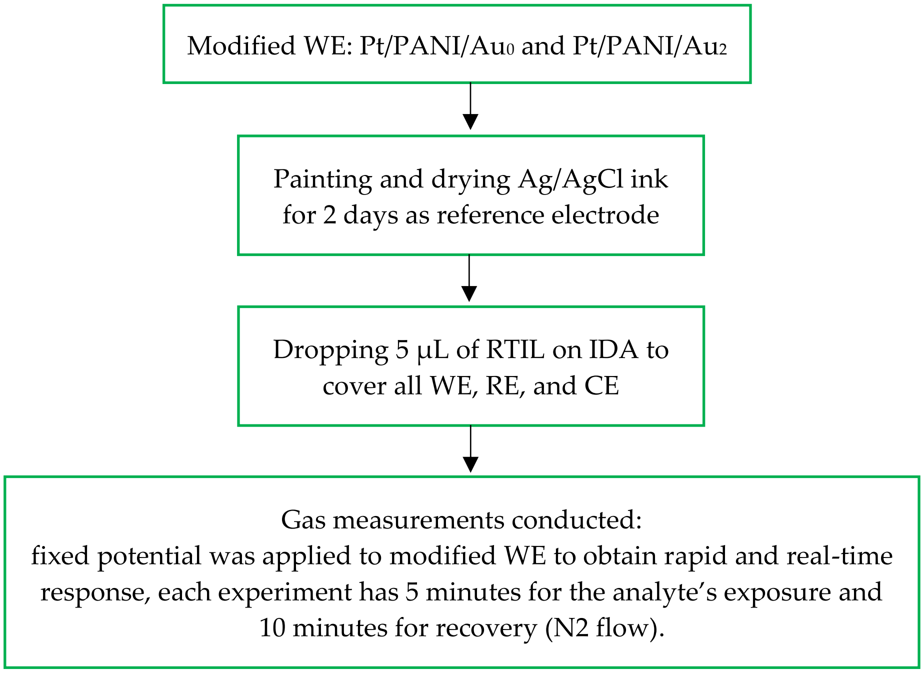

Supplement: Supplementary file 1 [file sensors-23-04132-s001.zip › sensors-2330617-supplementary.pdf]
